# Short-Term Differentiation of Glioblastoma Stem Cells Induces Hypoxia Tolerance

**DOI:** 10.1007/s11064-016-1868-2

**Published:** 2016-02-25

**Authors:** Håvard K. Skjellegrind, Artem Fayzullin, Erik. O. Johnsen, Lars Eide, Iver A. Langmoen, Morten C. Moe, Einar O. Vik-Mo

**Affiliations:** 1 Vilhelm Magnus Laboratory for Neurosurgical Research, Department of Neurosurgery and Institute for Surgical Research, Oslo University Hospital, Oslo, Norway; 2 Center for Eye Research, Department of Ophthalmology, Oslo University Hospital, Oslo, Norway; 3 Department of Medical Biochemistry, University of Oslo, Oslo, Norway

**Keywords:** Brain cancer stem cell, Hypoxia tolerance, Differentiation, Glioblastoma, GSC, Anoxia

## Abstract

Glioblastoma is the most common and malignant brain cancer. In spite of surgical removal, radiation and chemotherapy, this cancer recurs within short time and median survival after diagnosis is less than a year. Glioblastoma stem cells (GSCs) left in the brain after surgery is thought to explain the inevitable recurrence of the tumor. Although hypoxia is a prime factor contributing to treatment resistance in many cancers, its effect on GSC has been little studied. Especially how differentiation influences the tolerance to acute hypoxia in GSCs is not well explored. We cultured GSCs from three patient biopsies and exposed these and their differentiated (1- and 4-weeks) progeny to acute hypoxia while monitoring intracellular calcium and mitochondrial membrane potential (ΔΨ_m_). Undifferentiated GSCs were not hypoxia tolerant, showing both calcium overload and mitochondrial depolarization. One week differentiated cells were the most tolerant to hypoxia, preserving intracellular calcium stability and ΔΨ_m_ during 15 min of acute hypoxia. After 4 weeks of differentiation, mitochondrial mass was significantly reduced. In these cells calcium homeostasis was maintained during hypoxia, although the mitochondria were depolarized, suggesting a reduced mitochondrial dependency. Basal metabolic rate increased by differentiation, however, low oxygen consumption and high ΔΨ_m_ in undifferentiated GSCs did not provide hypoxia tolerance. The results suggest that undifferentiated GSCs are oxygen dependent, and that limited differentiation induces relative hypoxia tolerance. Hypoxia tolerance may be a factor involved in high-grade malignancy. This warrants a careful approach to differentiation as a glioblastoma treatment strategy.

**Electronic supplementary material:**

The online version of this article (doi:10.1007/s11064-016-1868-2) contains supplementary material, which is available to authorized users.

## Introduction

Glioblastoma multiforme (GBM) is the most common primary brain cancer. Current treatment combines surgery, radiation and chemotherapy. Median survival is less than 10 months in unselected patient groups [1]. The isolation of glioma cells possessing stem cell properties has shed new light on this cancer [2, 3]. Glioblastoma stem cells (GSCs) have shown resistant to both radio- and chemo-therapy [4–6]. GSCs left in the brain after tumor surgery is currently the leading theory explaining inevitable tumor recurrence [6]. Targeting the cells migrating from the tumor and surviving radiation and chemotherapy seems required for success. However, the true identity of these cells is not known. Chemo- and radio-resistance in gliomas is promoted by hypoxia [7] and the cells most resistant to treatment are found in the hypoxic parts of glioblastoma tumors [8]. Severe tumor hypoxia is associated with poor survival in both glioblastoma [9, 10] and several other cancers [11, 12]. It is therefore crucial to identify the cells that are most capable to survive in hypoxic conditions.

The effects of acute and chronic hypoxia on malignant cells can be different. Studies using cell culture in moderate chronic hypoxia (1–2 % O_2_) have shown enrichment of stem like cells in both glioma [13, 14] and other cancers [15, 16]. Acute, severe hypoxia (0–0.5 % O_2_) induces more cell death and thus strict selection of tolerant clones. Malignant progression has been found in many studies of acute tumor hypoxia, reviewed in [17]. Specifically, the histological features of glioblastoma including thrombosed vessels and pseudopallisading necrosis suggest that acute severe hypoxia has a central role in tumorigenesis [18]. Metabolic responses to acute hypoxia differ from that of chronic hypoxia [19]. Tolerance to acute hypoxia, however, is still a little explored phenomenon in cancer stem cells.

Differentiation is suggested as a potential treatment modality addressing GSCs [20]. However, in studies where immature cells have been transplanted to animals, both we and others have found better survival and integration of partially differentiated cells compared to undifferentiated cells [21–23]. The setting of transplantation itself is a hypoxic situation to the grafted cells, and tolerance to low oxygen supply could be important for survival and integration. Also in cancer metastasis, migrating cells would encounter low oxygen areas, and hypoxia tolerance could be important for the invasiveness of the cells. However, knowledge about hypoxia tolerance is limited. We have previously found increased resistance to hypoxia in immature brain cells [24]. The objective for this study was to investigate whether GCSs tolerate acute hypoxia and how differentiation influences their hypoxia tolerance.

## Methods and Materials

### Cell Culture

Glioblastoma biopsies were obtained from three informed and consenting patients during tumor surgery. Tissue harvesting was approved by the Norwegian Regional Committee for Medical Research Ethics (07321b). The biopsies were treated as previously described [25]. Briefly, the biopsies underwent mechanical and enzymatic dissociation before culture at 37 °C, 5 % CO_2_ in DMEM/F12 (Invitrogen) supplemented with 2 % B27 without retinoic acid (Invitrogen), 1 % HEPES (Lonza), 0.5 % heparin (Leo pharma), 100 U/ml streptomycin (Lonza), 100 U/ml penicillin (Lonza), 10 ng/ml bFGF (R&D Systems) and 20 ng/ml EGF (R&D Systems). For each passage, the cells were dissociated using trypsin (Invitrogen) before plating at 5.0 × 10^4^ cells/ml in 10 ml medium in 75 cm^2^ low adhesion flasks (Nunc). For induction of differentiation the medium contained DMEM/F12 with HEPES, Heparin, streptomycin, and penicillin as above, in addition to 4 % serum and 2 % B27 with retinoic acid, as previously described [26]. Glass slides and dishes were coated with Fibronectin 10 μg/ml (Sigma) in PBS. GSCs were differentiated for 1 or 4 weeks.

### Immunocytochemistry

GSCs were plated at 1600 cells/cm^2^ in fibronectin coated glass bottom dishes (WillCo-dish 40 mm, WillCo Wells). Undifferentiated cells were allowed 2 days to settle on the coated surface. Differentiated cells were fixed after 1 and 4 weeks. Cells were washed in PBS and fixed in 4 % paraformaldehyde. Permeabilization in 0.1 % Triton-X for 5 min before blocking with 5 % bovine serum albumin (BSA), 5 % donkey serum and 0.1 % Triton-X for 30 min. The primary antibodies reactive against βIII-tubulin (rabbit 1:1000, Sigma), nestin (goat 1:500), MAP-2 (mouse 1:500, Chemicon), GFAP (rabbit 1:1000, Dako), Ki-67 (mouse 1:1000) were diluted in PBS with 0.5 % BSA, 0.5 % donkey serum and 0.1 % Tween 20 and incubated overnight at 4 °C. Secondary antibodies, anti-mouse Alexa 488 (donkey 1:500, Invitrogen) anti-rabbit Alexa 555 (donkey 1:500, Invitrogen) and anti-goat Alexa 647 (donkey 1:500, Invitrogen), were incubated for 1 h at 20 °C. Nuclear staining was done using Hoechst 33258 (1:5000, Sigma).

### Fluorescence Imaging

Immunostained cells were imaged using an Olympus IX81 inverted fluorescence microscope equipped with excitation and emission filters for blue (ex: 350/50, em: 460/20), green (ex: 470/40, em: 515/30), red (ex: 545/30, em: 610/75) and deep red (ex: 620/60, em: 700/75). Images were acquired using Olympus soft imaging xcellance software. Post processing of the fluorescence images was done using the ImageJ package Fiji. All images presented for comparison were stained, imaged and processed identically.

### Time-Lapse Fluorescence Imaging

Cells were plated at a density of 500 cells/cm^2^ on fibronectin coated glass slides. To enable rapid exchange of medium, the slides were attached to a flow chamber. Perfusion fluid was artificial cerebrospinal fluid (ACSF) with the following composition (in mM): NaCl: 123; KCl: 3.75; KH_2_PO_4_: 1.25; NaHCO_3_: 26; dextrose: 10; MgCl_2_: 1 and CaCl_2_: 2 [27]. pH was controlled by CO_2_ perfusion. Before imaging the cells were incubated with 30 μM Rhodamine 123 (Rh123) to monitor mitochondrial membrane potential (ΔΨ_m_) [28] and 2 μM Fura-2/AM for Ca^2+^ imaging [29]. Time-lapse imaging was done using an Olympus IX81 inverted fluorescence microscope with temperature control. Rh123 was imaged using 492/18 nm excitation and 531/32 nm emission filters. Phototoxicity and photobleaching were reduced by using a 3.9 % neutral density (ND) filter. Fura-2 was excited at 340/15 and 380/15 nm (11 % ND) and recorded using a 510/40 emission filter. For hypoxia solutions, oxygen was removed by N_2_ perfusion before addition of an oxygen scavenger, sodium dithionite (0.75 mM; Sigma-Aldrich). Scavenger toxicity was ruled out by control experiments using oxygenated ACSF containing sodium dithionite. After hypoxia 1 μM Carbonyl cyanide 4-(trifluoromethoxy) phenylhydrazone (FCCP; Sigma) was used to totally depolarize the mitochondria [30], and thereby demonstrate any preserved ΔΨ_m_ by increase in Rh123 fluorescence. 5 μM Ionomycin (Sigma) was used as a positive control of the [Ca^2+^]_i_ recording. The cells were scored as lost or preserved ΔΨ_m_ and stable or unstable [Ca^2+^]_i_. Increase of the slope of Rh123 fluorescence after FCCP was used as the criterion for scoring preserved ΔΨ_m_. Non-reversed increase of Fura-2 ratio within the hypoxia period was used as criteria for unstable [Ca^2+^]_i_.

### Computerized Analysis of Fluorescence Images

Ki-67 labeled cells were quantified using the open source image analysis software CellProfiler [31]. Hoechst and Ki-67 images were thresholded. Nuclei (Hoechst) and Ki-67 positive nuclei were identified as primary objects. Hoechst and Ki-67 labeling were related as parent and child objects. Ki-67 positive and negative cells were thereby counted using the same criteria in both undifferentiated, 1 week differentiated and 4 weeks differentiated cultures. ≥4 experiments and more than 600 cells were counted in each experimental group.

### Gene Expression Analyses

Undifferentiated and differentiated cells were washed in PBS twice. The cells were further lysed by QiAzol (Qiagen) and stored at −20 °C. Total RNA was extracted using TRIzol Reagent according to the manufacturer’s instructions (Life Technologies). RNA concentration and purity were measured using Nanodrop (Wilmington, DE). Reverse transcription was performed using the High Capacity cDNA Reverse Transcription Kit (Life Technologies), with 300 ng total RNA per 20 μl reaction volume. qRT-PCR was performed using the StepOnePlus RT-PCR system (Life Technologies) and TaqMan Gene Expression Assays following protocols from the manufacturer (Life Technologies). The thermo cycling conditions were 95 °C for 10 min followed by 40 cycles of 95 °C for 15 s. and 60 °C for 1 min. The data were analyzed using the 2^−ΔΔCt^ method as fold change relative to control, using GAPDH as endogenous control. All samples were run in duplicates. TaqMan Gene Expression assays include GFAP (Hs00909233_m1), NES (Hs00707120_s1), GAPDH (Hs99999905_m1), β-III-tubulin (Hs00801390_s1), and CD133 (Hs01009250-m1). Results are presented as fold change. Error bars are R-min/-max.

### Oxygen Consumption and Acidification Rate

Cells in all three groups of differentiation state were plated on SeaHorse 24 well plates at a density of 5.0 × 10^4^ cells/well 1 day before experiments. Medium was changed to buffer-free DMEM containing 2 mM glutamine, 5 mM pyruvate and 10 mM glucose 1 h before the start of the flux analyses. Simultaneous analyses of oxygen consumption rate (OCR) and extracellular acidification rate (ECAR) were performed on a Seahorse XF24-3 extracellular flux analyzer (Seahorse Bioscience). After baseline measurements, the following compounds were added with subsequent measurements: oligomycin (2 µg/ml), 2 additions of FCCP (1 µM), FCCP, rotenone (0.1 µM). Oxidative/glycolytic ratio was calculated by dividing average oxygen consumption rate (OCR) by extracellular acidification rate (ECAR). To ensure a valid comparison of OCR and ECAR, the cell numbers were confirmed by nuclear staining (as described under immunolabeling) after the flux measurements. Automated counting of cells after fluorescence microscopy was done as described above.

### Flow Cytometry

Flow cytometry was performed using an Accuri C6 flow cytometer. Live single cells were gated using forward and side scatter. From this population of cells mean fluorescence values were used. ΔΨ_m_ was measured using the ratiometric mitochondrial dye JC-1 [32]. Mitochondrial mass was quantified in methanol fixed cells using the fluorescent dye Nonyl Acridine Orange, that binds to cardiolipin in the inner mitochondrial membrane independently on energetic state [33] and with a linear relationship between dye incorporation and cardiolipin content [34]. Analysis of cytometry data was performed using FloJo software.

### Statistics

Rh123 fluorescence data were normalized and Fura-2 ratios calculated using MS Excel. Mean values from each experiment were used for statistics. The data were transferred to GraphPad Prism 6.0 for statistics and presentation. ANOVA was used for statistical analysis of difference between groups. The hypoxia experimental groups consisted of at least five experiments each. Other experiments were conducted in triplicates or more. Results were considered significant where *p* < 0.05.

## Results

Experiments were performed using three different glioblastoma stem cell cultures obtained from three patients at primary surgical treatment without any previous oncological treatment. The three GSC cultures are referred to as T1, T2 and T3. Cells demonstrated self-renewal by exponential growth for 20 passages. The maintenance of tumorigenicity and expression of stem cell markers was maintained upon culturing under sphere-forming conditions [3, 25, 35].

**Fig. 1:**
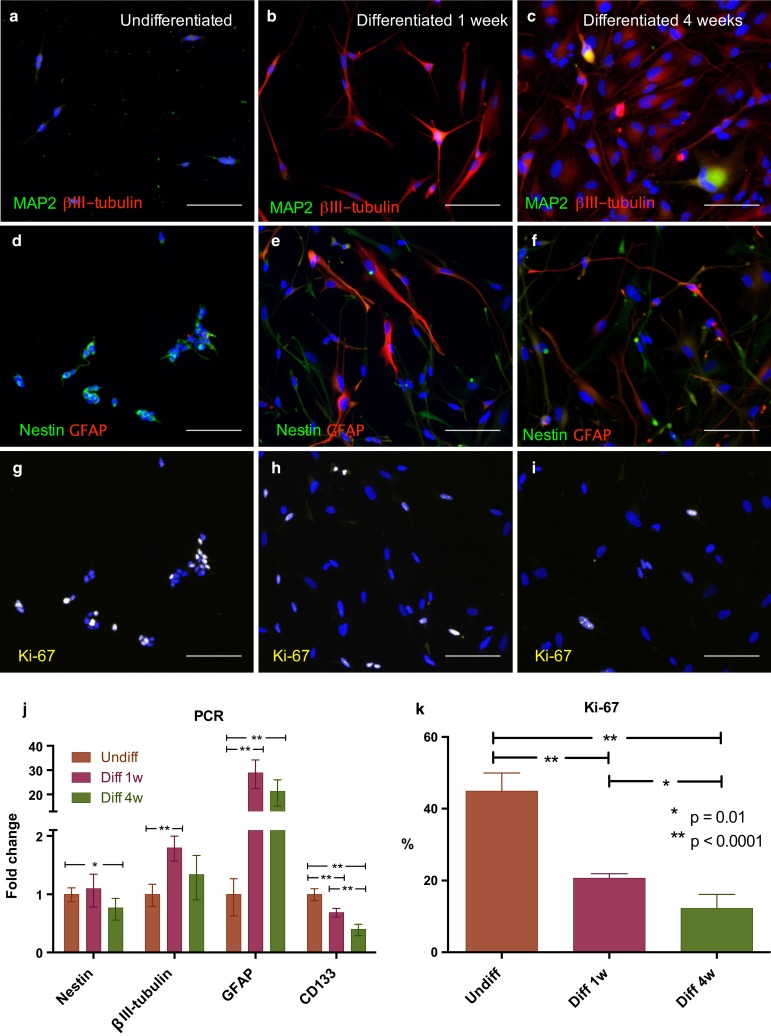
GSCs differentiation induced a more mature phenotype and reduced proliferation. Undifferentiated GSCs had highly positive staining of the neuronal stem cell marker nestin. Differentiation led to development of both neural and glial phenotype, shown by the neuronal marker βIII-tubulin and glial marker GFAP. After 4 weeks differentiation a few cells also expressed the mature neuronal marker MAP-2. Proportions of immuno-reactive cells assessed by manual counting: **a** undifferentiated GSCs: MAP2 0 %; βIII-tubulin 52 % (weak staining). **b** 1 week differentiated GSCs: MAP2 0.7 %; βIII-tubulin 95 %. **c** 4 weeks differentiated GSCs: MAP2 2 %; βIII-tubulin 89 %. **d** undifferentiated GSCs: nestin 97 %; GFAP 6 %. **e** 1 week differentiated GSCs: nestin 41 %; GFAP 29 %. **f** 4 weeks differentiated GSCs: nestin 28 %; GFAP 25 %. **g**–**i** Proliferation was reduced, shown by a lower proportion of Ki-67 positive cells after differentiation (Ki-67 positive nuclei shown as white due to merge with blue nuclear stain). *Blue*: Hoechst in all images. *Scale bar* 100 μm. **j** Gene expression analyzed by qRT-PCR of undifferentiated, 1 week differentiated and 4 weeks differentiated GSC cultures. βIII-tubulin was significantly up-regulated after 1 week differentiation, GFAP expression was highly increased after differentiation. Nestin expression was significantly reduced after 4 weeks differentiation. CD133 expression was significantly reduced with increasing differentiation. The *bars* represent experiments in triplicate or more. **k** Computerized counting of Ki-67 positive cells from a representative GSC culture. N = 4 experiments and more than 600 cells in each differentiation state

Differentiation was assessed morphologically by immunolabeling and quantitatively by qPCR (Fig. 1). Immunocytochemistry displayed decreasing levels of the immature stem cell marker nestin, and to development of both neural and glial phenotype, shown by the neuronal marker βIII-tubulin and glial marker GFAP. After 4 weeks differentiation a few cells also expressed the mature neuronal marker MAP-2. Ki-67, a marker of cellular proliferation was found in 45.0 % (38.5–51.6) of undifferentiated cells, 18.9 % (12.9–24.8) in 1 week and 11.6 % (6.2–17.1) in 4 weeks differentiated cells. In qPCR nestin expression was found significantly reduced after 4 weeks differentiation (*p* < 0.05). The GSC marker CD133 expression was reduced at 1 week and further reduced at 4 weeks differentiation (both *p* < 0.01). βIII-tubulin expression increased significantly from undifferentiated state to 1 week differentiated (*p* < 0.01). GFAP expression at 1-week differentiation was increased 28-fold compared to undifferentiated and still high at 4 weeks differentiation (21-fold).

**Fig. 2:**
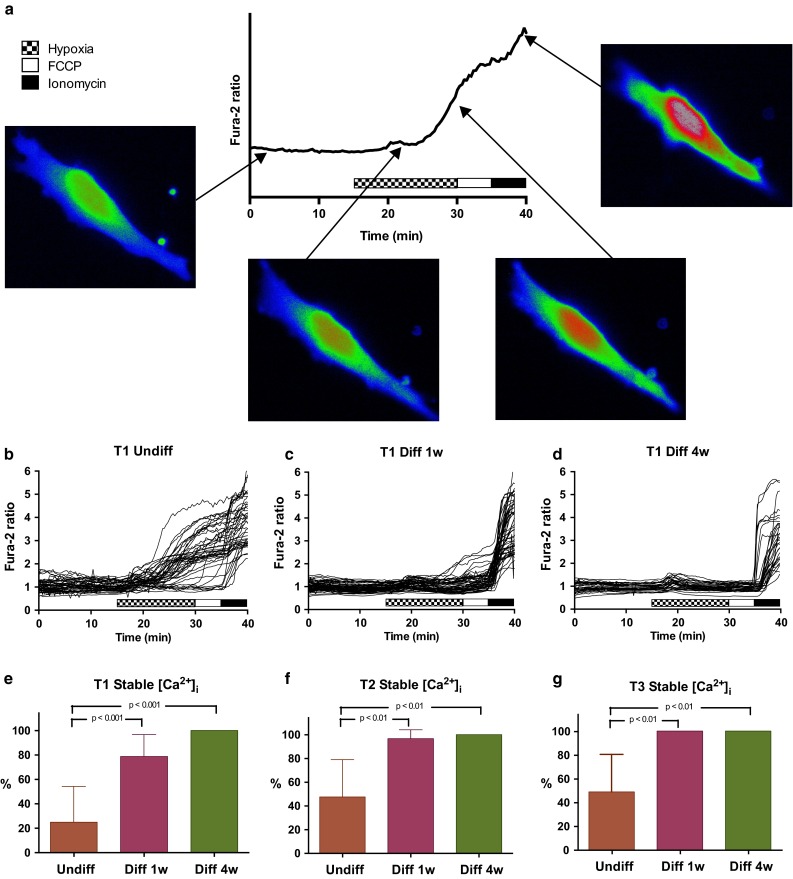
Ca^2+^ stability during hypoxia was improved after 1 week differentiation and sustained at 4 weeks. **a** Schematic presentation of a hypoxia experiment. Intracellular calcium change was recorded using Fura-2 staining and fluorescence time-lapse microscopy. Presented images are pseudocolor images of fura-2 ratio. Baseline recording show stable calcium levels. Calcium increase during hypoxia is shown by increased Fura-2 ratio. Application of Ionomycin at the end of experiments confirms the ability to detect calcium changes. Fura-2 ratio measurements of **b** undifferentiated, **c** 1 week differentiated and **d** 4 week differentiated GSCs. For graphs of all three tumors, see Online Resource 1. **e**–**g** Proportions of cells that maintained stable intracellular calcium during 15 min hypoxia were significantly increased from undifferentiated to differentiated state in all three GSC cultures, which represent cells from three different patient samples. In all groups n ≥ 5 experiments and ≥25 cells

Calcium deregulation is a widely used early indicator of neuronal death induced by ischemic stress [36]. In animals, reduced calcium response to hypoxia is regarded as a component of hypoxia tolerance [37]. As an indicator of hypoxia tolerance, we used the ability to maintain intracellular calcium integrity during hypoxia. This was measured by monitoring changes in fluorescence from the calcium indicator Fura-2. Figure 2a shows a representative trace of an undifferentiated GSC with Fura-2 ratio images from the different phases of the experiment. Figure 2b–d show traces of the Fura-2 ratio for each of the cells of the T1 tumor in experiments of undifferentiated (b), 1 week differentiated (c) and 4 weeks differentiated cells (d). The tumors T2 and T3 are shown in Online Resource 1. The proportions of cells maintaining stable [Ca^2+^]_i_ are shown for all three tumor cultures in Fig. 2e–g. Based on reports of hypoxia tolerance in immature neural cells [24, 38] and GSC enrichment in hypoxic tumor areas [8] we hypothesized that the most immature cells would tolerate hypoxia the best. The majority of undifferentiated GSCs, however, suffered from a sustained increase in [Ca^2+^]_i_. The proportions of cells maintaining stable [Ca^2+^]_i_ in the three undifferentiated cultures were T1: 25.0 % (−11.4 to 61.3), T2: 47.7 % (14.8–80.5) and T3: 48.9 % (9.9–88.0; Fig. 2e–g). Thus, only a minority of the undifferentiated GSCs was able to maintain stable intracellular calcium during acute hypoxia.

A far lower proportion of differentiated GSCs revealed a [Ca^2+^]_i_ increase that did not return to baseline level during hypoxia. 78.8 % (60.0–97.5) of T1 cells maintained stable calcium. For T2 and T3 the proportions were 96.7 % (87.4–105.9) and 100 % (100–100) respectively (Fig. 2e, f). Stability of [Ca^2+^]_i_ was significantly better in the 1 week differentiated cells compared to the undifferentiated cells in all tumors tested (T1 *p* < 0.001, T2 and T3 *p* < 0.01, ANOVA).

From all three cultures of 4 weeks differentiated GSCs, all cells maintained intracellular calcium during the 15 min hypoxia period. Some of the cells had a small but transient increase, and then went back to baseline level. Ionomycin induced a twofold to threefold increase in Fura-2 ratio in all cells, demonstrating valid [Ca^2+^]_i_ measurements. The difference compared to undifferentiated GSCs was significant for all three tumors (T1 *p* < 0.001, T2 and T3 *p* < 0.01, ANOVA).

**Fig. 3:**
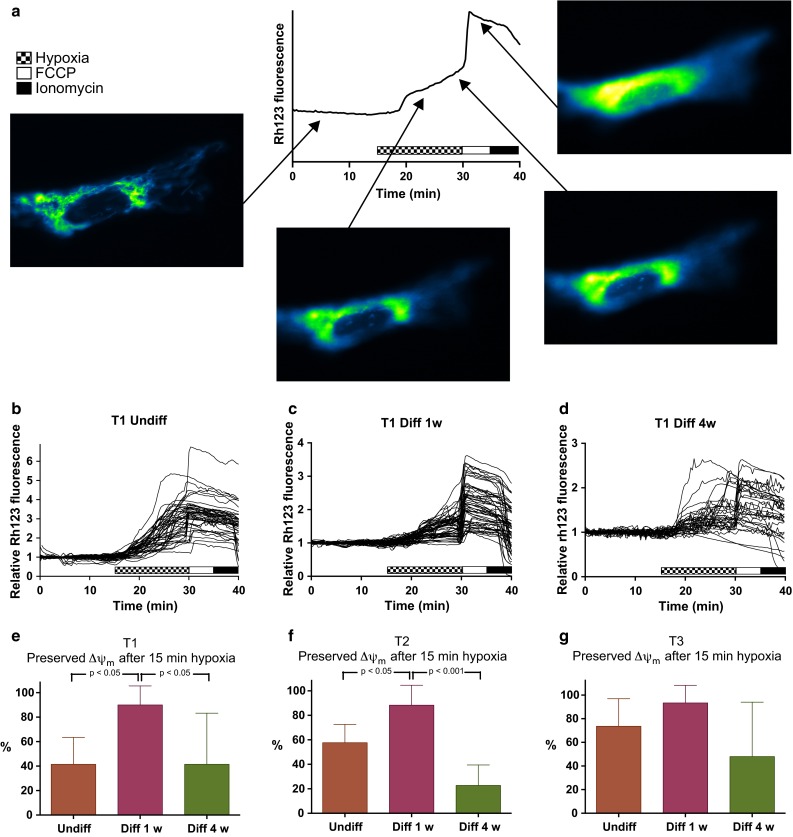
Mitochondrial hypoxia tolerance was improved only at 1 week differentiation. **a** Schematic presentation of a hypoxia experiment. Baseline recording shows stable ΔΨ_m_. During hypoxia the fluorescence increases due to mitochondrial depolarization. After FCCP remaining ΔΨ_m_ is depolarized shown by further increase in fluorescence before decrease due to diffusion of rh123 out of the cell. **b**, **c**, **d** Traces of Rhodamine 123 fluorescence in the same cells as in Fig. 2 showing depolarization of mitochondrial membrane potential upon hypoxia and FCCP in **b** undifferentiated, **c** 1 week differentiated and **d** 4 week differentiated GSCs. The proportion of cells showing preserved ΔΨ_m_ after hypoxia was in **b** undifferentiated GSCs 41.4 % (14.1–68.8), **c** 1 week differentiated GSCs 90.0 % (73.6–106.3) and **d** 4 weeks differentiated GSCs 41.4 % (2.8–80.0). For graphs of all three tumors, see Online Resource 2. **e**–**g** The *bar graphs* show for the different three tumors the proportion of cells that had preserved mitochondrial membrane potential after 15 min hypoxia, with significant increased hypoxia tolerance development from undifferentiated to 1 week differentiated state in and reversal of this to 4 weeks differentiation in tumor cultures T1 and T2. In all groups n ≥ 5 experiments and ≥25 cells

Another early sign of fatal damage to brain cells is loss of mitochondrial membrane potential (ΔΨ_m_). Mitochondrial depolarization is an early step in programmed cell death and also a downstream event of temozolomide treatment [8], which is standard chemotherapy for glioblastoma patients. Mitochondrial function is thus a relevant indicator of hypoxia tolerance. The Warburg theory postulates independence of oxidative ATP production in cancer tissue. The dependence on mitochondrial energy metabolism in GSCs is not well studied. Before experimental hypoxia, the cells were incubated with both Fura-2 and the ΔΨ_m_ sensitive dye Rhodamine 123 (Rh123) and thus changes in ΔΨ_m_ could also be monitored. Figure 3a shows a representative recording of ΔΨ_m_ during hypoxia in an undifferentiated GSC. Figure 3b–d show the traces of Rh123 fluorescence from the single cells in the experiments on GSC culture T1. T2 and T3 are shown in Online Resource 2. The proportions of cells that preserved ΔΨ_m_ during hypoxia are shown in Fig. 3e–g. A large proportion of the undifferentiated cells exhibited early loss of mitochondrial membrane potential during hypoxia. This was shown by increase in cytoplasmic Rh123 fluorescence due to release of dye accumulated in the mitochondria (Fig. 3b). After the hypoxia period, FCCP was applied to determine which cells had remaining mitochondrial membrane potential. A further increase in fluorescence indicates that mitochondrial membrane potential was still to some degree preserved at this point. The proportion of cells showing remaining ΔΨ_m_ after hypoxia in undifferentiated GSCs was for T1 41.4 % (14.1–68.8), T2 57.6 % (41.9–73.4) and T3 73.6 % (44.6–102.6) (Fig. 3e–g).

One week differentiated GSCs also responded to 15 min hypoxia by some mitochondrial depolarization (Fig. 3c). However only a few of the cells were totally depolarized. One week differentiated cells with a preserved ΔΨ_m_ represented for T1 90.0 % (73.6–106.3), T2 88.3 % (68.2–108.5) and T3 93.3 % (74.8–111.8). Compared to the undifferentiated cells, the fraction of cells with complete loss of mitochondrial membrane potential were significantly lower among the 1 week differentiated GSCs of T1 and T2 (*p* < 0.05, ANOVA). The same trend was seen in T3, but not statistically significant.

**Fig. 4:**
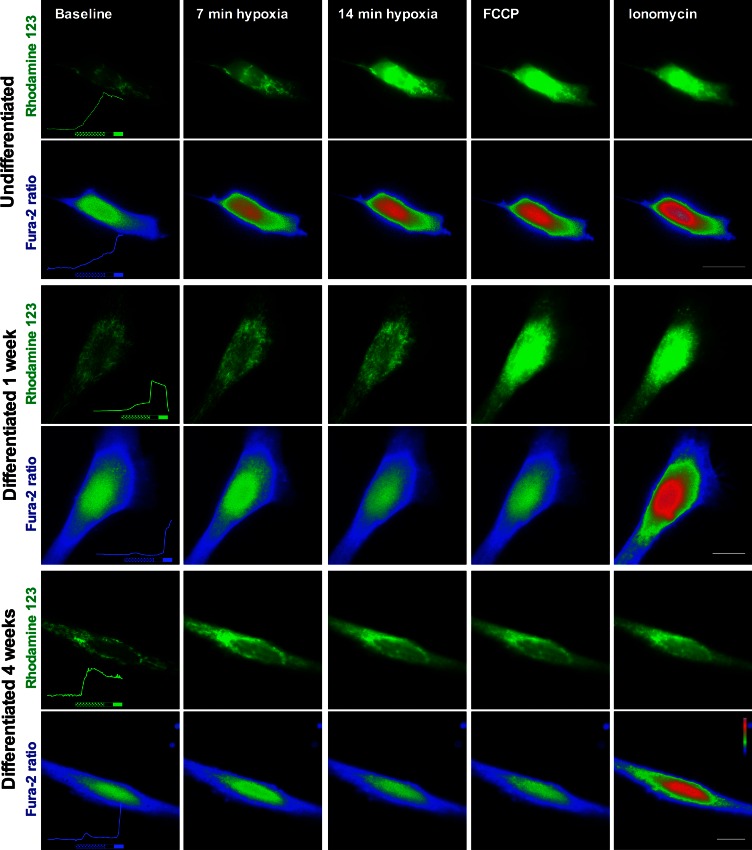
Representative time-lapse fluorescence microscopy images of undifferentiated, 1 week differentiated and 4 weeks differentiated GSCs exposed to 15 min hypoxia. Fura-2 is presented as pseudocolor ratio images. Traces of Rh123 fluorescence and Fura-2 ratio in the actual cells are presented as inset in the first images. Hypoxia, FCCP and Ionomycin are marked as in Figs. 2 and 3. Increasing fluorescence of Rh123 indicates mitochondrial membrane depolarization. Increasing fura-2 ratio indicates increase of intracellular calcium levels. The undifferentiated cell lost mitochondrial membrane potential and control of intracellular calcium during 15 min hypoxia. There was no reaction to FCCP on mitochondrial membrane potential. The 1 week differentiated cell mitochondria are partially depolarized during hypoxia, but shows preserved potential by increase in Rh123 fluorescence at FCCP application. Intracellular calcium is stable through the hypoxic period. The 4 weeks differentiated cell has fully depolarized mitochondria after 15 min hypoxia, shown by no increase in Rh123 fluorescence after FCCP application. Intracellular calcium is stable during hypoxia. In all cells intracellular calcium increased after release of extracellular calcium into the cell by ionomycin. *Scale bars* 20 μm

In GSCs differentiated for 4 weeks (Fig. 3d) Rh123 fluorescence increased during hypoxia, demonstrating depolarization of the mitochondrial membrane. The majority of 4 week differentiated T1 and T2 GSCs did not preserve any mitochondrial membrane potential after 15 min hypoxia. In addition, the cells from T3 showed the same pattern of development compared to 1 week differentiated cells, however not statistically significant. The 4 weeks differentiated GSCs preserved mitochondrial membrane potential after 15 min hypoxia in 41.4 % (2.8–80.0) of T1, 22.8 % (5.2–40.3) of T2 and 48.0 % (−9.2 to 105.2) of T3 (Fig. 3e–g). In two of the tumors the mitochondrial hypoxia tolerance was significantly lowered compared to 1 week differentiation (T1 *p* < 0.05, T2 *p* < 0.001, ANOVA). Representative time-lapse fluorescence images of undifferentiated, 1 week differentiated and 4 weeks differentiated GSCs are shown in Fig. 4 and Online resources 3, 4 and 5.

**Fig. 5:**
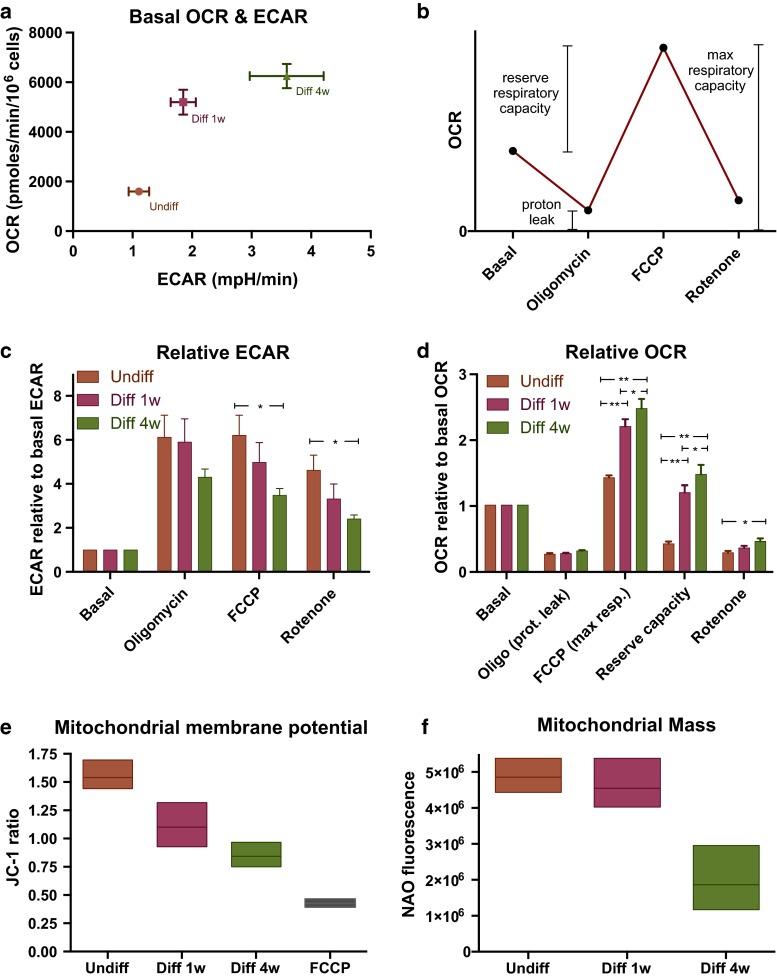
Respiration, ΔΨ_m_ and mitochondrial mass were changed by differentiation. Oxygen consumption rate (OCR) and extracellular acidification rate (ECAR) were measured simultaneously in cultures of undifferentiated, 1 and 4 weeks differentiated GSCs. The measurements were adjusted to actual cell count. **a** Basal metabolism show low absolute OCR and ECAR in undifferentiated cells, and increasing metabolic rate with increasing differentiation. **b** Schematic presentation of the events and measurements in the experiments: OCR after blocking of mitochondrial complex V by oligomycin shows the oxygen consumption used due to proton leak across the inner mitochondrial membrane. Reserve respiratory capacity is the difference between max respiration induced by FCCP and basal level of oxygen consumption. **c**, **d** ECAR and OCR measurements normalized to baseline. **e** ΔΨ_m_ in undifferentiated and differentiated GSCs shown as JC-1 ratio measured by flow cytometry. ΔΨ_m_ was the highest in undifferentiated cells and decreased with increasing differentiation. FCCP treated cells as control. **f** Mitochondrial mass was assessed by Nonyl Acridine Orange and flow cytometry, and found similar in undifferentiated and 1 week differentiated cells, but decreased after 4 weeks differentiation. Arbitrary fluorescence units on the Y-axis. All experiments were performed at least three times

After finding increased tolerance to hypoxia in 1 week differentiated cells, we wanted to investigate whether differentiation changed the energy metabolism of the cells. As seen in Fig. 5a, undifferentiated GSCs had a low basal rate of oxygen consumption and acidification rate. OCR and ECAR were increased after both 1 and 4 weeks differentiation. However, the OCR/ECAR ratio was not significantly different between the groups. Further measurements were normalized to baseline values in order to enable comparison between the groups. Oligomycin was added to irreversibly block the mitochondrial ATP synthase/ATPase. Both production and consumption of ATP by the mitochondria were thus inhibited. The values recorded following this treatment are measures of the oxygen consumption needed to compensate for proton leakage across the inner mitochondrial membrane. The proton leak relative to basal metabolism was not significantly different between undifferentiated and differentiated cells.

FCCP treatment leads to uncoupling and thereby maximal oxygen consumption in the mitochondria. The difference between maximal and basal oxygen consumption is termed the reserve respiratory capacity (Fig. 5b). The relative reserve respiratory capacity was the highest in 4 week differentiated cells, and significantly less in 1 week differentiated and least in undifferentiated cells. The increased respiration capacity is due to enhanced complex I activity (Fig. 5d). Inhibition of mitochondrial ATP production by oligomycin combined with FCCP led to more increase in relative glycolysis (ECAR) in undifferentiated GSCs compared to 4 weeks differentiated cells (Fig. 5c).

In order to further characterize the mitochondrial function in undifferentiated and differentiated GSCs resting mitochondrial membrane potential was investigated. The mitochondrial membrane potential is high when activity is low and reduced if activity is high. In order to reduce bias due to differences in cell size, the ratiometric two color fluorescent probe JC-1 was chosen. Red and green fluorescence was measured by flow cytometry and the red/green ratio compared (Fig. 5e). ΔΨ_m_ was significantly different when comparing undifferentiated cells to both 1 week (*p* < 0.05) and 4 weeks (*p* < 0.01) differentiated cells. The difference between our one and 4 weeks differentiated cells was not statistically significant. JC-1 ratio of FCCP treated cells was used as a control of totally depolarized cells and was found significantly lower compared to all other groups.

The low oxygen consumption and lower maximal respiration in undifferentiated cells suggested a possible difference in mitochondrial contents in the cells. To compare mitochondrial mass in undifferentiated and differentiated GSCs we stained fixed cells from each group with Nonyl Acridine Orange (NAO). NAO stains cardiolipin in the inner mitochondrial membrane in a linear relationship to mitochondrial membrane surface area and can thus be used as an indicator of mitochondrial mass in the cell. Mitochondrial mass was found similar in undifferentiated and 1 week differentiated cells (no significant difference). Four week differentiated cells had significantly less NAO fluorescence compared to both undifferentiated and 1 week differentiated cells (both *p* < 0.01), indicative of less mitochondrial mass in the long-term differentiated cells (Fig. 5f).

## Discussion

The main findings of this work were (a) undifferentiated GSCs did not tolerate acute hypoxia, (b) limited differentiation induced tolerance to acute hypoxia and (c) mitochondrial hypoxia tolerance was lost after prolonged differentiation. The lack of hypoxia tolerance in our most immature cells was not expected. Experiments on the neonatal brain have previously demonstrated hypoxia tolerance in immature brain cells [24, 38]. Based on this and previous reports of reduced mitochondrial activity in stem cells [39] and cancer cells [40–42], the most immature of our cells were expected to be the least oxygen dependent, and thus the most hypoxia tolerant. However, our results suggest that the most immature GSCs are oxygen dependent. This can be seen in coherence with the perivascular localization of GSCs in tumors [43]. The Warburg effect, i.e. impaired oxidative metabolism in cancer tissue [42], has been suggested evident in GSCs [44]. Our measurements of oxygen consumption (OCR) and lactate generation (ECAR; Fig. 5a) revealed a comparable relationship between oxidative and glycolytic metabolism in both undifferentiated and differentiated cells. Undifferentiated cells had the least relative reserve respiratory capacity (Fig. 5d), indicating a basal oxidative metabolism almost at maximum. Even though undifferentiated cells had the same mitochondrial mass as 1 week differentiated cells (Fig. 5f), the absolute basal oxygen consumption was significantly lower (Fig. 5a). Low absolute oxygen consumption in the most immature cells is in agreement with the findings of other groups [40, 45, 46], but also contrary to a recent study using a GBM cell line [47]. In controlled experiments there is an inverse proportional relationship between ΔΨ_m_ and respiration rate [48]. In our experiments, the highest ΔΨ_m_ was found in the undifferentiated cells (Fig. 5e). The high ΔΨ_m_ corresponds with the low basal oxygen consumption relative to mitochondrial mass found in the undifferentiated cells. This finding could fit with the Warburg theory of inhibited oxidative metabolism. However, the oxygen dependency and lack of increased glycolytic metabolism suggests a reason for doubt on the Warburg effect in these GSCs.

A majority of the GSCs subjected to 1 week of differentiation stimuli developed a tolerance to acute hypoxia. These cells were able to preserve both calcium homeostasis and mitochondrial membrane potential during 15 min acute hypoxia. Maintenance of ΔΨ_m_ without oxygen is a process where the mitochondrial ATP synthase is reversed and consumes ATP to maintain a proton gradient across the inner mitochondrial membrane [49]. This is possible as long as ATP is available and the ΔΨ_m_ is not disrupted by other factors, such as the mitochondrial pore transition (MPT) [50], which can be induced by calcium overload. The 1 week differentiated cells did not show signs of calcium overload. The amounts of ATP needed to maintain ΔΨ_m_ without oxygen is dependent on the proton leak across the inner mitochondrial membrane. Such leakage is dependent upon the level of the ΔΨ_m_ and the expression of uncoupling proteins. Proton leak increase with increasing ΔΨ_m_. The ΔΨ_m_ obtained by ATP synthase reversal is in the magnitude of approximately −80 mV, while in normal respiration ΔΨ_m_ is about −150 mV [51]. The proton leak at −80 mV is relatively low. Our data on basal metabolism show similar relative proton leak in the undifferentiated and differentiated cells (Fig. 5d), indicating similar expression of uncoupling proteins. The finding of a superior mitochondrial hypoxia tolerance in the 1 week differentiated cells suggests that these cells have the best ability to maintain all ATP needs by non-oxidative metabolism. This is consistent with oligomycin sensitivity in GSC sphere culture, but not in adherent serum stimulated GSC derived cells [52]. Hypoxia tolerance is also found in glioblastoma cell line U-87 after induction to express the neural progenitor protein doublecortin (DCX) [53]. Transplantation of cells to the brain is a known hypoxic situation. In a previous study from our lab, predifferentiation of neural stem cells before transplantation to mice produced improved survival and integration of the graft [21]. Similarly a study on transplantation of embryonal and post-natal retinal cells found the best results using cells of limited maturation [22].

The 4 weeks differentiated cells did not show mitochondrial hypoxia tolerance. However, intracellular calcium was stable in all cells. This could indicate that in these cells, the mitochondria are fragile, but other cell functions can be maintained without oxidative ATP production. Basal ΔΨ_m_ was the lowest in this group. Some depolarization is seen in cells with very active mitochondrial metabolism [48]. It is also seen as a property of aging [54]. The absolute oxygen consumption was slightly higher in 4 weeks differentiated cells compared to 1 week, even though mitochondrial mass was less (Fig. 5f). This group also had the least relative increase in glucose utilization after inhibition of mitochondrial ATP-production by oligomycin (Fig. 5c). An already high dependence upon glycolytic metabolism could contribute to the increased hypoxia tolerance found in terms of stable intracellular calcium during hypoxia. Whether the mitochondrial depolarization seen during hypoxia in this group induces any permanent damage is not clarified by this study, and needs further investigation. In hypoxia tolerant animals and neonatal mice, hypoxia induces a small and temporary increase of intracellular calcium [37], and this has been suggested as indicative of cell survival instead of late hypoxic death through apoptosis. Such calcium fluctuation has also been suggested as a signal in hypoxic preconditioning [55]. A small and temporary increase in intracellular calcium after 3–5 min hypoxia was seen in many of our differentiated GSCs (Fig. 2c, d, and Online Resource 1).

The in vitro culture conditions are always a possible confounder of results that are interpreted as relevant to true in vivo properties. However, our culture protocol does maintain stem cell properties, multipotency and tumorigenicity through multiple passages [25]. In contrast to traditional cancer cell lines, often cultured in as much as 10 % serum, our undifferentiated cells did not get serum supplements. Serum free culture conditions seem important for maintenance of tumor stem cell (TSC) properties [35]. Culture in ambient oxygen level is the common method, although ambient oxygen is actually hyperoxia to cells. Still, the cells preserve TSC properties [25]. To challenge cells with acute hypoxia we have used 0 % oxygen. Hypoxia experiments are often performed using 1–2 % oxygen levels. This is, however, closer to the physiologic oxygen tension, and especially in tumors low oxygen levels are common. Close to zero oxygen is needed for relevant acute hypoxia effects [56].

The clinical relevance of tumor hypoxia on CSCs is related to treatment resistance and malignant progression. Hypoxia dependent temozolomide resistance was recently shown in GSCs [46]. Differentiation has been suggested as a possible GBM treatment strategy [20]. Whether partially differentiated GSCs possess a malignant potential is uncertain. However, Ki-67 immunoreactivity in some of these cells (Fig. 1h) indicates a sustained proliferative ability [57]. A mechanism of bidirectional plasticity may exist. Induced reprogramming resulting in glioma propagating capacity has recently been shown in both mouse neurons [58] and differentiated GSCs [59].

Although having low basal oxygen consumption, our undifferentiated GSCs did not show hypoxia tolerance compared to partially differentiated cells of the same origin. Prolonged differentiation impaired mitochondrial hypoxia tolerance. The results of this study suggest that partly differentiated rather than undifferentiated GSCs possess the ability to utilize the therapy resistance offered by tissue hypoxia. This also implies that treatment of tumor hypoxia and hypoxia related resistance could be insufficient to eradicate GSCs. The knowledge on the malignancy potential of partially differentiated GSCs is still sparse. This needs elucidation before development of in vivo differentiation therapy for glioblastoma.

## Electronic supplementary material

Below is the link to the electronic supplementary material.

Online Resource 1 Single cell traces of Fura-2 ratio in undifferentiated and differentiated GSCs exposed to 15 min hypoxia, 5 min FCCP and 5 min Ionomycin. In undifferentiated GSCs stable [Ca^2+^]_i_ was maintained in **a** 25.0% (-11.4-61.3), **b** 47.7% (14.8-80.5) and **c** 48.9% (9.9-88.0) of the cells. In one week differentiated GSCs from tumor T1, T2 and T3 the proportions of cells maintaining stable [Ca^2+^]_i_ were **d** 78.8% (60.0-97.5), **e** 96.7% (87.4-105.9) and **f** 100% (100-100). **g**, **h** & **i** In four weeks differentiated cells [Ca^2+^]_i_ was maintained stable in all cells from all three tumors (PDF 235 kb)

Online Resource 2 Single cell traces of normalized Rh123 fluorescence in undifferentiated and differentiated GSCs exposed to 15 min hypoxia, 5 min FCCP and 5 min Ionomycin. The proportions of cells with preserved ΔΨ_m_ after 15 min hypoxia in undifferentiated GSCs from tumors T1, T2 and T3 were **a** 41.4% (14.1-68.8) **b** 57.6% (41.9-73.4) **c** 73.6% (44.6-102.6). In one week differentiated GSCs preserved ΔΨ_m_ was found in **d** 90.0 % (73.6-106.3), **e** 88.3% (68.2-108.5) and **f** T3 93.3 % (74.8-111.8). The four weeks differentiated GSCs preserved ΔΨ_m_ after 15 min hypoxia in **g** 41.4% (2.8-80.0), **h** 22.8% (5.2-40.3) and **i** 48.0% (-9.2-105.2) (PDF 285 kb)

Online Resource 3 Representative time-lapse fluorescence microscopy video of an undifferentiated GSC. The left image represents Rh123 staining of mitochondria. The right image is a pseudocolor presentation of Fura-2 ratio. Increasing Rh123 fluorescence indicates mitochondrial membrane depolarization. Increasing fura-2 ratio (shift from blue to green to red) indicates increased [Ca^2+^]_i_. This undifferentiated cell lost mitochondrial membrane potential and control of intracellular calcium during 15 min hypoxia. There was no reaction to FCCP on the ΔΨ_m_. Ionomycin leads to release of extracellular calcium into the cell and thus increased Fura-2 ratio (AVI 2257 kb)

Online Resource 4 Representative time-lapse fluorescence microscopy video of a one week differentiated GSC. The left image represents Rh123 staining of mitochondria. The right image is a pseudocolor presentation of Fura-2 ratio. Increasing Rh123 fluorescence indicates mitochondrial membrane depolarization. Increasing fura-2 ratio (shift from blue to green to red) indicates increased [Ca^2+^]_i_. In this one week differentiated cell the mitochondria are partially depolarized during hypoxia but still show preserved ΔΨ_m_ by increase in Rh123 fluorescence at FCCP application. Intracellular calcium is stable through the hypoxic period. Ionomycin leads to release of extracellular calcium into the cell and thus increased Fura-2 ratio (AVI 2392 kb)

Online Resource 5 Representative time-lapse fluorescence microscopy video of a four weeks differentiated GSC. The left image represents Rh123 staining of mitochondria. The right image is a pseudocolor presentation of Fura-2 ratio. Increasing Rh123 fluorescence indicates mitochondrial membrane depolarization. Increasing fura-2 ratio (shift from blue to green to red) indicates increased [Ca^2+^]_i_. This four weeks differentiated cell has fully depolarized mitochondria after 15 min hypoxia, shown by no increase in Rh123 fluorescence after FCCP application. Intracellular calcium is stable during hypoxia. Ionomycin leads to release of extracellular calcium into the cell and thus increased Fura-2 ratio (AVI 1896 kb)
