# Supplementary figures and images for: Short-Term Differentiation of Glioblastoma Stem Cells Induces Hypoxia Tolerance
Source: Neurochem Res. 2016 Feb 25;41(7):1545–58. doi: 10.1007/s11064-016-1868-2 (PMC4893075; doi:10.1007/s11064-016-1868-2)

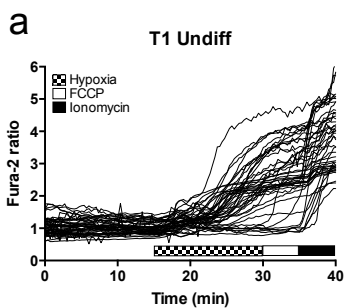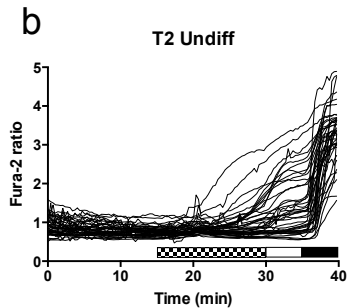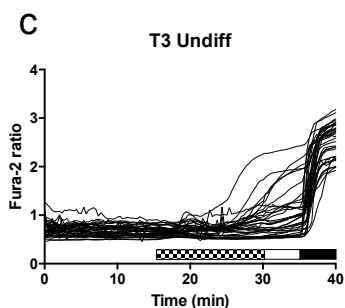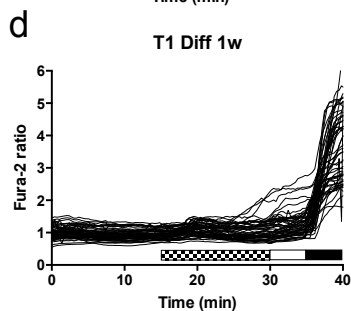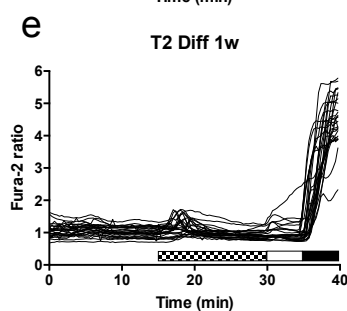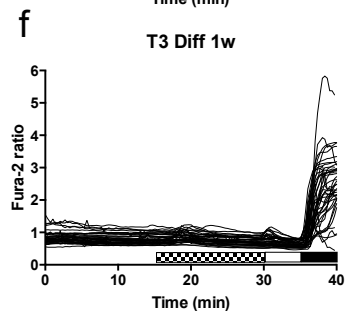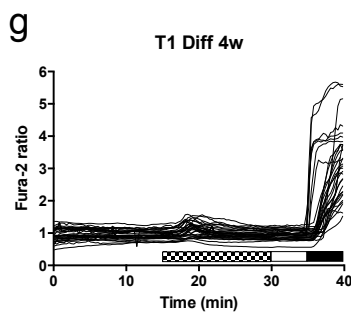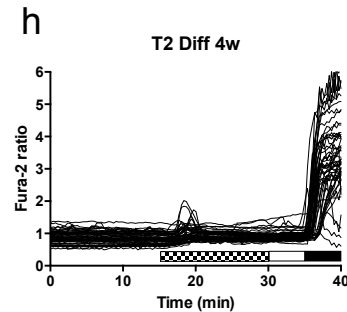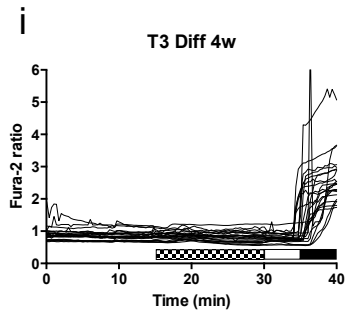

Supplement: Supplementary file 1 — Single cell traces of Fura-2 ratio in undifferentiated and differentiated GSCs exposed to 15 min hypoxia, 5 min FCCP and 5 min Ionomycin. In undifferentiated GSCs stable [Ca2+]i was maintained in a 25.0% (-11.4-61.3), b 47.7% (14.8-80.5) and c 48.9% (9.9-88.0) of the cells. In one week differentiated GSCs from tumor T1, T2 and T3 the proportions of cells maintaining stable [Ca2+]i were d 78.8% (60.0-97.5), e 96.7% (87.4-105.9) and f 100% (100-100). g, h & i In four weeks differentiated cells [Ca2+]i was maintained stable in all cells from all three tumors (PDF 235 kb) [file 11064_2016_1868_MOESM1_ESM.pdf]

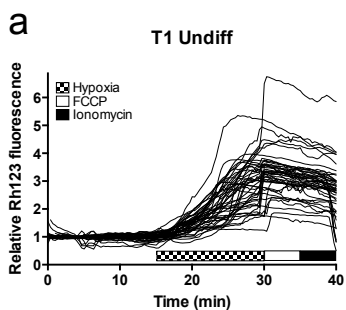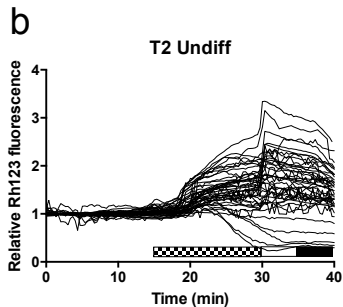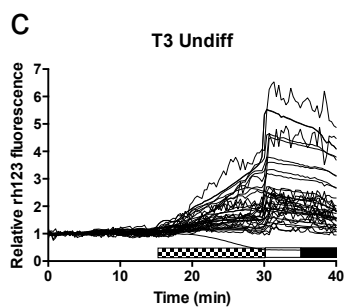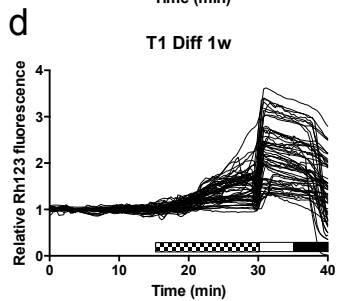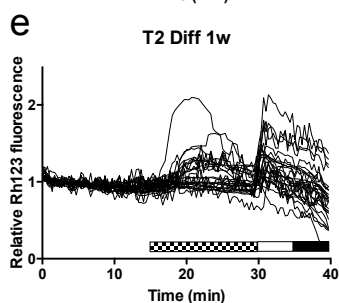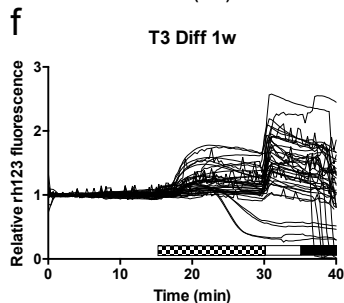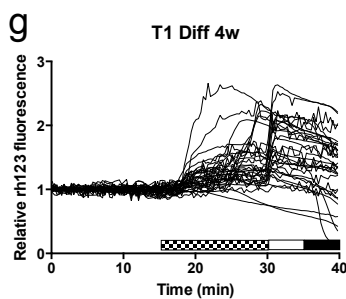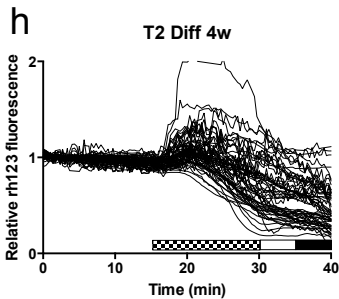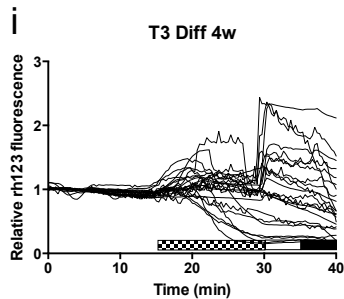

Supplement: Supplementary file 2 — Single cell traces of normalized Rh123 fluorescence in undifferentiated and differentiated GSCs exposed to 15 min hypoxia, 5 min FCCP and 5 min Ionomycin. The proportions of cells with preserved ΔΨm after 15 min hypoxia in undifferentiated GSCs from tumors T1, T2 and T3 were a 41.4% (14.1-68.8) b 57.6% (41.9-73.4) c 73.6% (44.6-102.6). In one week differentiated GSCs preserved ΔΨm was found in d 90.0 % (73.6-106.3), e 88.3% (68.2-108.5) and f T3 93.3 % (74.8-111.8). The four weeks differentiated GSCs preserved ΔΨm after 15 min hypoxia in g 41.4% (2.8-80.0), h 22.8% (5.2-40.3) and i 48.0% (-9.2-105.2) (PDF 285 kb) [file 11064_2016_1868_MOESM2_ESM.pdf]
